# Supplementary material for: LtGAPR1 Is a Novel Secreted Effector from Lasiodiplodia theobromae That Interacts with NbPsQ2 to Negatively Regulate Infection
Source: J Fungi (Basel). 2023 Jan 31;9(2):188. doi: 10.3390/jof9020188 (PMC9967411; doi:10.3390/jof9020188)
Supplement: Supplementary file 1 [file jof-09-00188-s001.zip › jof-2184705-supplementary.pdf]

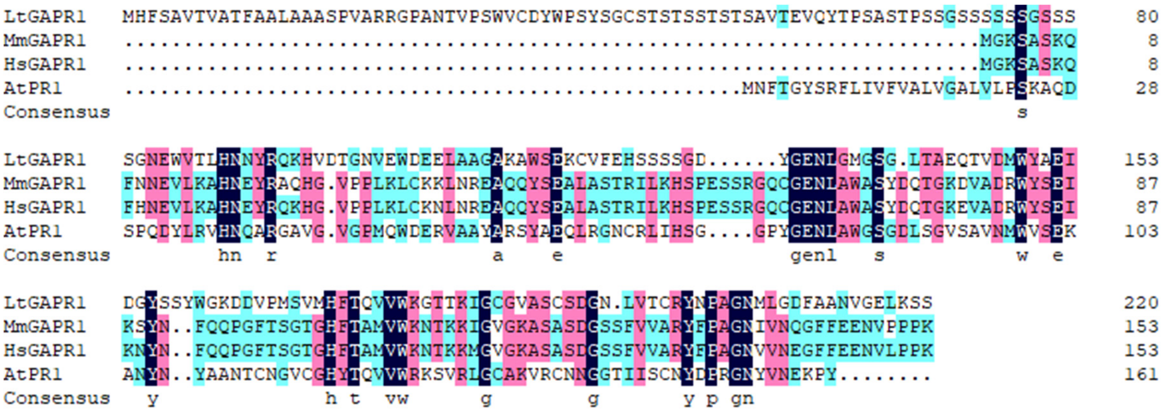

Figure S1. alignment of the LtGAPR1 protein with the PR-1 proteins in other species.

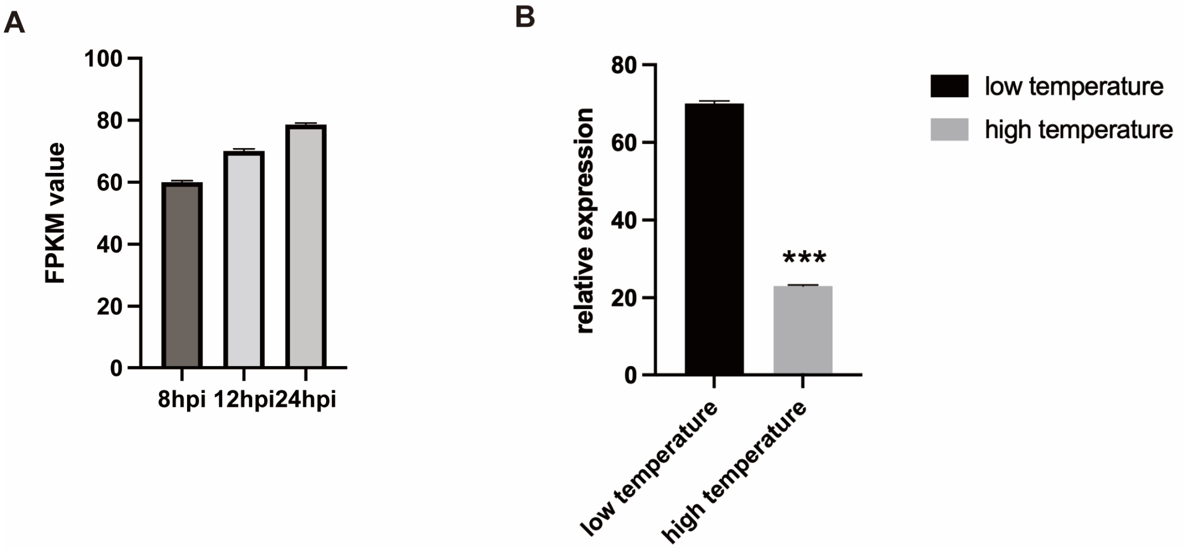

Figure S2. the gene expression of *LtGAPR1* in early infection of *L. theobromae* and the gene expression of *LtGAPR1* in different temperature.

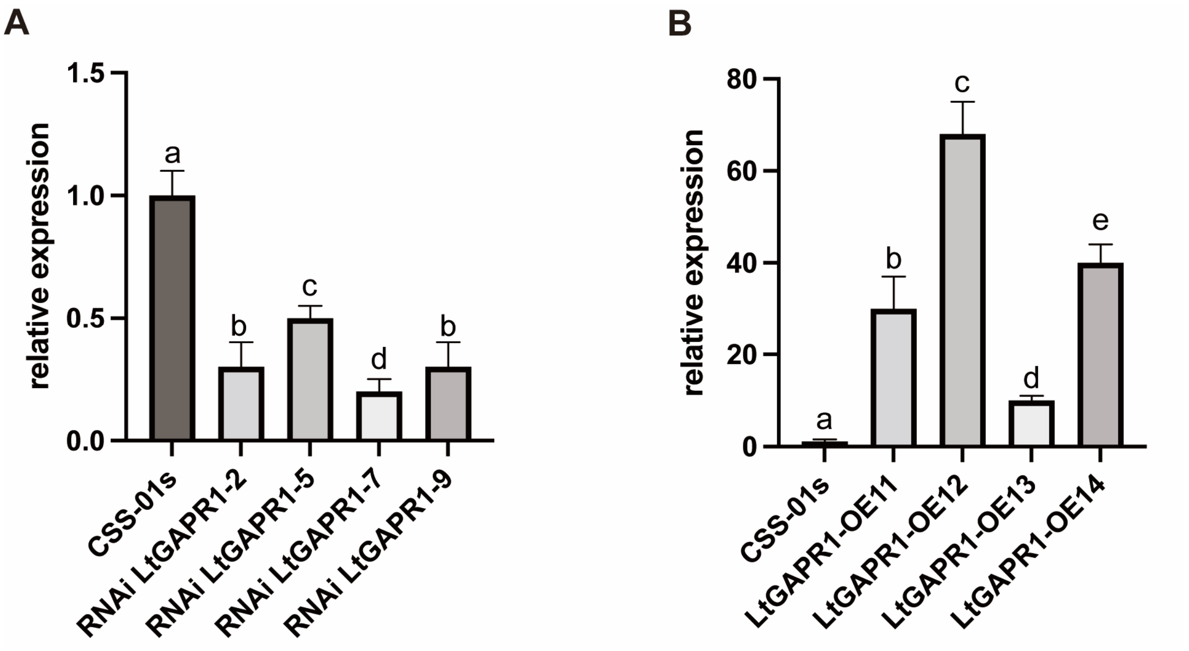

Figure S3. the relative gene expression of *LtGAPR1* in overexpression and silenced transformants.

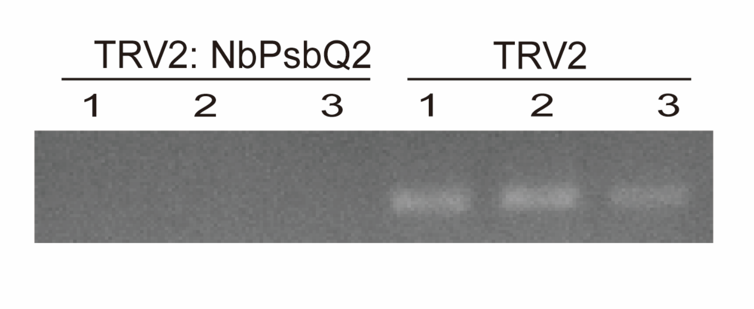

Figure S4. the gene expression of *NbPsbQ2* after silencing were detected by RT-PCR.

Table S1. primers used in this research.

Name	primer 5' to 3'
vigs-NbPsbQ2-F	AAGGAGATTGTTAGCGTCAAG
vigs-NbPsbQ2-R	TTAACCAAGTTTGGCCAAAAC
OE-LtGAPR1-F	ATGCATTTCTCTGCTGTGACT
OE-LtGAPR1-R	CTACGAGCTGGACTTGAGCTC
RNAi-LtGAPR1-F	AAGTGTGTGTTTGAGCACTCT
RNAi-LtGAPR1-R	GCTGTAGCCGTCGATCTCGGC
